# Early-age strength evolution and brittle-to-ductile transition mechanism of basalt-fiber-reinforced cemented gangue backfill

**DOI:** 10.1038/s41598-026-46049-0

**Published:** 2026-03-26

**Authors:** Jinfeng Mao, Xiaoming Shi, Jianye Feng, Yikun Liu, Xiaodong Zheng, Lixia Zhu, Chenhui Liu, Hengfeng Liu

**Affiliations:** 1 School of Mining Engineering and Geology, Xinjiang Institute of Engineering, Urumqi, 830023 China; 2 Key Laboratory of Xinjiang Coal Resources Green Mining, Ministry of Education, Xinjiang Institute of Engineering, Urumqi, 830023 China; 3 School of Energy and Mining Engineering, China University of Mining and Technology-Beijing, Beijing, 100083 China; 4 Xinjiang Key Laboratory of Coal-bearing Resources Exploration and Exploitation, Xinjiang Institute of Engineering, Urumqi, 830023 China; 5 Xinjiang Engineering Research Center of Green Intelligent Coal Mining, Xinjiang Institute of Engineering, Urumqi, 830023 China; 6 Jinjie Coal Mine, CHN Energy, Yulin, 719319 China; 7 School of Mines, China University of Mining and Technology, Xuzhou, 221116 China

**Keywords:** Basalt fiber, Cemented gangue backfill, Early-age strength, Failure-mode regulation, Uniaxial compressive strength, Crack evolution, Engineering, Materials science

## Abstract

High brittleness and rapid post-peak capacity loss are often observed in cemented gangue backfill (CGB) under uniaxial compression. The risk of sudden instability in deep mining panels is therefore increased. Chopped basalt fibers (0-0.60 wt%) were incorporated to improve early-age load capacity and ductility, and curing ages of 3–60 d were investigated. Uniaxial compression tests were performed. Crack evolution was interpreted mainly through Acoustic emission (AE) characteristics and surface failure observations, with digital image correlation DIC serving as an auxiliary qualitative tool. Scanning electron microscopy and discrete element simulations were used to clarify multiscale toughening mechanisms. An optimal dosage was identified at 0.3 wt%. At 28 d, uniaxial compressive strength (UCS) and peak strain were increased by 67.2% and 37.0%, reaching 5.71 MPa and 3.7%. A rapid strength-gain window occurred from 3 d to 7 d. For the 0.3 wt% mixture, UCS rose from 0.74 MPa to 3.23 MPa (436.49%), supporting mining initiation at approximately 7 d after backfilling. At 7 d, tensile cracks dominated the reference mixture (82.6%). After fiber addition, crack deflection and branching were promoted and shear sliding was activated. At 0.3 wt%, the shear-associated crack proportion increased markedly, while crack deflection and branching were promoted. Failure mode evolved from concentrated longitudinal splitting toward a more distributed oblique mixed-mode crack network, and post-peak softening was mitigated. A dense C-S-H-rich hydration layer was formed in situ on fiber surfaces, creating a fiber-hydration product-matrix interface. Strength and damage tolerance were jointly improved through bridging-based load transfer, stress redistribution, and crack-tip blunting. The results clarify the early-age strengthening and quasi-brittle toughening mechanism of basalt-fiber-reinforced cemented gangue backfill and provide a basis for mixture optimization and early-age support design.

## Introduction

Cemented gangue backfill (CGB) has been increasingly adopted in underground mining because it can simultaneously achieve goaf support, solid-waste utilization, and environmental-impact mitigation^1–3^. After slurry placement and hardening, the backfill mass participates in load bearing, improves the stress environment of the panel, restrains overburden movement and surface subsidence, and supports safer resource recovery under appropriate conditions^4–7^.

Despite these advantages, conventional CGB still exhibits pronounced quasi-brittle behavior under compression, characterized by low tensile resistance, limited deformation capacity, and rapid post-peak strength loss^8–13^. Because the backfill must begin to accommodate mining-induced stress redistribution soon after placement, reliable service performance depends not only on compressive strength but also on sufficient resistance to cracking and deformation. Therefore, improving early-age load-bearing development and suppressing brittle failure remain key challenges in gangue-based backfill systems^14–17^.

CGB is a composite material composed of solid-waste aggregates and cementitious binders, and its structural and mechanical characteristics show certain similarities to those of concrete^18^. Under uniaxial compression, CGB specimens commonly exhibit tensile-dominated failure, accompanied by high brittleness, insufficient ductility, and relatively low peak strain. As a result, the practical demand for stable load bearing and deformation resistance in the panel is not fully satisfied^19,20^. To address this limitation, the incorporation of an appropriate amount of fibers into backfill mixtures has been demonstrated to be an effective toughening approach, by which strength can be improved and resistance to cracking, bending, and impact can be enhanced to varying degrees^21,22^. With increasing fiber dosage, an overall upward trend in strength is typically observed, and the failure mode can shift gradually from pure tensile failure to a combined tension-shear pattern, indicating more stable post-peak load-bearing behavior^23^. AE monitoring has been widely used to identify fracture activity, damage evolution, and stage-dependent instability in brittle geomaterials. Recent studies on granite under compression and cyclic loading-unloading have shown that AE activity, energy dissipation, and damage evolution exhibit strong coupling during the progressive failure process, and that AE descriptors can effectively capture transitions from crack initiation to peak and post-peak degradation^24,25^. At the microscale, scanning electron microscopy (SEM) observations have shown that abundant hydration products can adhere to fiber surfaces and form a relatively dense interfacial structure with the matrix. Crack coalescence is thus delayed through a bridging effect, and crack resistance is improved^26^. Fiber reinforcement is an effective strategy for mitigating crack concentration and improving deformation coordination in cement-based materials. Among the available fiber types, basalt fiber is particularly attractive because of its high tensile strength, high elastic modulus, favorable fracture resistance, and good compatibility with cementitious matrices. When an appropriate amount is used, basalt fiber can bridge developing cracks, restrain crack propagation, and improve the resistance of the matrix to unstable fracture^27,28^. By comparison, basalt fiber is characterized by high strength and elastic modulus, favorable fracture toughness, and good compatibility with cementitious systems, and advantages in improving crack resistance and flexural performance have been reported^29^. When an appropriate amount is used, cracks can be effectively bridged, crack propagation and fissure development can be suppressed, and tensile as well as impact resistance can be enhanced^30^.

Existing studies on basalt fiber have been conducted predominantly in conventional civil-engineering cementitious materials, whereas its toughening mechanism in underground cemented gangue backfill remains insufficiently resolved. This knowledge gap is particularly important because gangue-based backfill differs from ordinary cement-based composites in terms of aggregate heterogeneity, pore structure, weak interfacial regions, and failure-path complexity. In particular, three issues remain unclear in gangue-based backfill systems. First, the role of basalt fiber in regulating strength build-up and deformation coordination during the early-age support window has not been systematically clarified, although this stage directly controls whether the backfill can provide reliable initial roof support. Second, it remains uncertain whether the observed improvement should be interpreted merely as strength enhancement or as a multiscale toughening process involving crack deflection, delayed crack coalescence, and redistribution of fracture modes in a highly heterogeneous gangue-rich matrix. Third, the intrinsic linkage among fiber bridging, fiber-hydration interfacial evolution, acoustic-emission-based crack activity, and mesoscale fracture-network development has not yet been established in an integrated manner.

To address the engineering challenge of high brittleness and unstable post-peak load-bearing behavior in CGB, basalt fiber was introduced for toughening and reinforcement, and a combined investigation integrating laboratory tests and discrete element simulations was conducted. A comparative matrix was established with fiber dosages of 0-0.6 wt% and curing ages of 3–60 d. The evolution of strength, deformation response, failure mode, and crack propagation characteristics was systematically analyzed for early-age and middle-to-late stages. By correlating macroscopic mechanical indices with microscale crack evolution, the mechanism by which basalt fiber content enhances the mechanical system of the backfill was elucidated. The findings provide verifiable material evidence and engineering reference for mixture proportion optimization, early load-bearing control, and safe support design in goaf areas using basalt-fiber-reinforced gangue backfill.

## Materials and methods

Chopped basalt fibers with a length of 6 mm were used in the experiments. The main mineral constituents were dominated by oxides such as SiO_2_, Al_2_O_3_, and CaO, and the key mechanical parameters are listed in Table 1. This fiber length was selected by considering both crack-bridging effectiveness and dispersion stability in the gangue-based slurry matrix. Fibers that are too short are less effective in bridging developing microcracks and restraining crack coalescence, whereas excessively long fibers are more prone to entanglement, non-uniform distribution, and local weak zones in a highly heterogeneous backfill system.

To evaluate the influence of fibers on the mechanical response of CGB, five dosage levels were designed based on the mass fraction of basalt fiber relative to the total mass of solid materials, namely 0%, 0.15%, 0.30%, 0.45%, and 0.60%. A CGB slurry system was adopted. Specimens were prepared by adding basalt fibers at different dosages into a mixed slurry composed of gangue, fly ash, and cement. The slurry concentration was set to 75%, and the mass fractions of cement, fly ash, and gangue were 10%, 20%, and 45%, respectively; the detailed quantities for each mixture are provided in Table 2. Based on the five fiber dosages, five curing ages were selected, including 3 d, 7 d, 14 d, 28 d, and 60 d. For each curing age, three parallel specimens were prepared for each fiber dosage to ensure the stability and comparability of the test results.

Because basalt fiber was incorporated as part of the total solids, a slight adjustment in water dosage was introduced with increasing fiber content to maintain the target slurry concentration of 75% and ensure comparable workability among mixtures.

**Table 1:** Mechanical properties of basalt fibers.

Item	Length (mm)	Density (g/cm^3^)	Diameter (µm)	Tensile strength (MPa)	Elastic modulus (GPa)	Ultimate elongation (%)
Basalt fiber	6	2.65	17	1050	100	3

**Table 2:** Mixture proportions of cemented gangue backfill with different basalt fiber contents.

Fiber dosage (wt%)	Gangue (kg)	Fly ash (kg)	Cement (kg)	Water (kg)	Basalt fiber (kg)	Curing age (d)
0.00	0.65	0.289	0.1445	0.3613	0	3/7/14/28/60
0.15	0.65	0.289	0.1445	0.3618	0.001625	3/7/14/28/60
0.30	0.65	0.289	0.1445	0.3623	0.003251	3/7/14/28/60
0.45	0.65	0.289	0.1445	0.3634	0.004876	3/7/14/28/60
0.60	0.65	0.289	0.1445	0.364	0.006502	3/7/14/28/60

According to the mix proportions in Table 2, gangue, cement, fly ash, tap water, and basalt fibers were weighed separately using a high-precision electronic balance. The backfill slurry was homogenized with an automatic mixer and then poured sequentially into cylindrical molds that had been pre-coated with dimethyl silicone oil. The molds were cylinders with a diameter of 50 mm and a height of 100 mm. After 24 h of curing, demolding was conducted. The demolded backfill specimens remained geometrically stable and were labeled and transferred to a constant-temperature and constant-humidity curing chamber for curing periods of 3 d, 7 d, 14 d, 28 d, and 60 d. The curing temperature was maintained at 21 °C with a relative humidity of 96%.

**Fig. 1:**
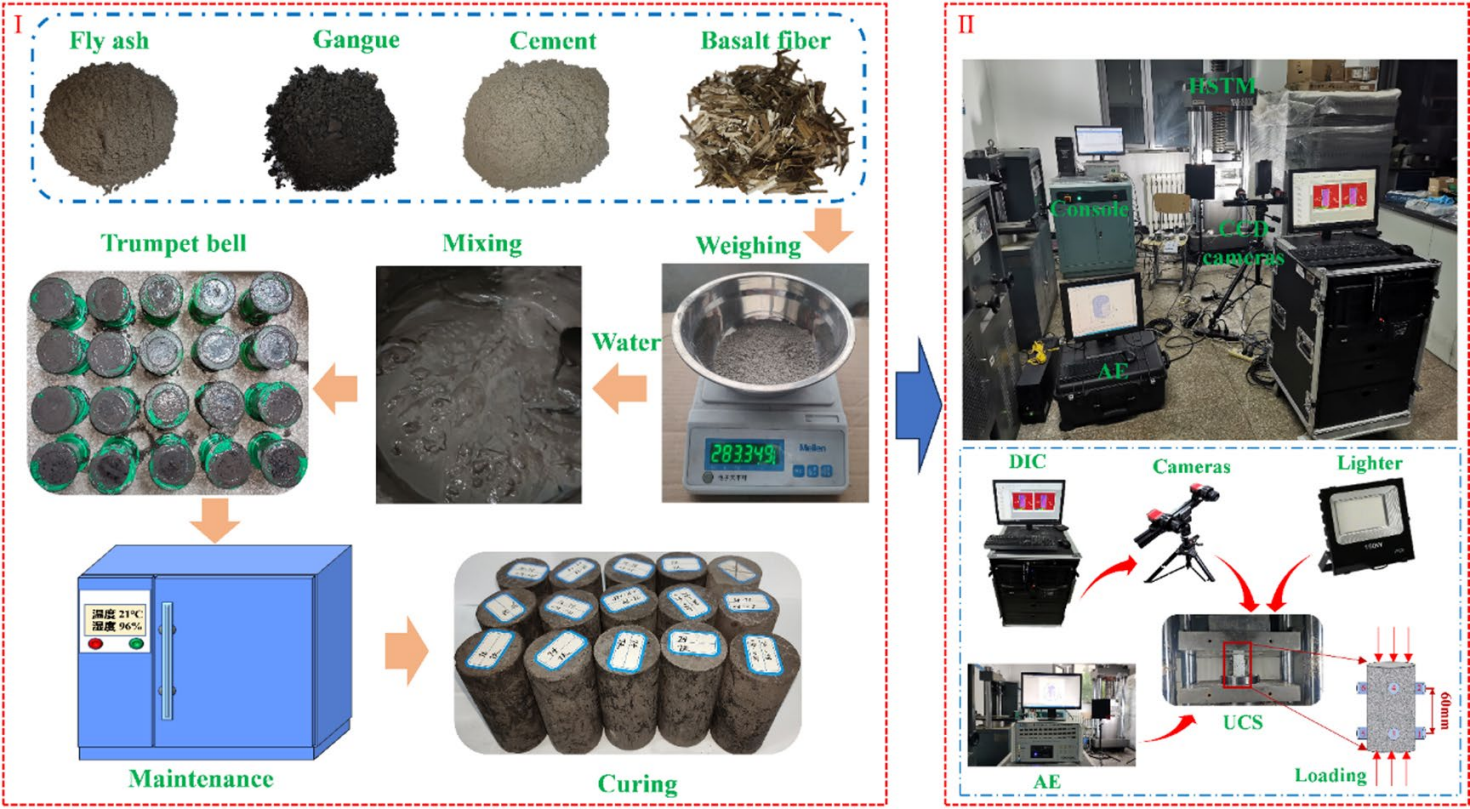
Flow chart of the specimen preparation and the space arrangement of the experimental system.

When the target curing age was reached, the specimens were removed for mechanical testing. Uniaxial compression tests were conducted using a YAD-2000 hydraulic servo universal testing machine under displacement control until failure, and the complete stress-strain response was recorded during loading. To assist the interpretation of damage evolution and surface deformation characteristics, acoustic emission monitoring and three-dimensional DIC observation were synchronously performed during selected compression tests. A commercial AE acquisition system was used to record the evolution of acoustic activity during loading, and a three-dimensional DIC system was employed to capture the surface deformation and failure development of the specimens. In addition, scanning electron microscopy was used to examine the interfacial morphology and hydration-product distribution in representative samples after testing. The experimental procedure is shown in Fig. 1.

## Results

### Effect of basalt fiber content on the mechanical properties of CGB

**Fig. 2:**
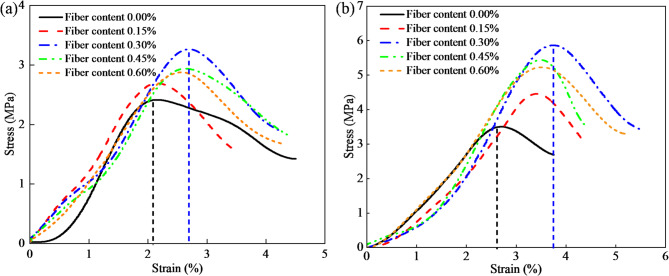
Stress-strain curve of CGB with different basalt fiber contents. (**a**) 7d, (**b**) 28d.

The incorporation of basalt fiber markedly improved not only the peak stress and peak strain of CGB, but also the overall stress-strain response, as shown in Fig. 2. The fiber-free specimens exhibited a relatively sharper post-peak stress drop, indicating a stronger brittle failure tendency. After basalt fiber incorporation, the pre-peak deformation capacity increased and the post-peak stress drop became less abrupt, suggesting that fiber bridging delayed the coalescence of dominant cracks and improved deformation coordination. As the fiber dosage increased from 0 to 0.60 wt%, the maximum compressive strength generally increased first and then decreased, indicating the existence of an optimal dosage range for this system. At 0.30 wt%, the 7 d specimens exhibited the best performance, with a compressive strength of 3.23 MPa and a peak strain of 2.68%, representing increases of 33.6% and 27.8%, respectively, compared with the fiber-free group. After curing to 28 d, these values further increased to 5.71 MPa and 3.7%, corresponding to improvements of 67.2% and 37.0%, respectively. However, a further increase in fiber dosage weakened the improvement, likely because excessive fibers reduced the structural uniformity of the matrix. Overall, an appropriate basalt fiber dosage improves both the load-bearing capacity and the resistance to sudden post-peak instability under uniaxial compression.

### Effect of curing age on the mechanical properties of basalt-fiber-reinforced CGB

#### Evolution of compressive strength and peak strain

**Fig. 3:**
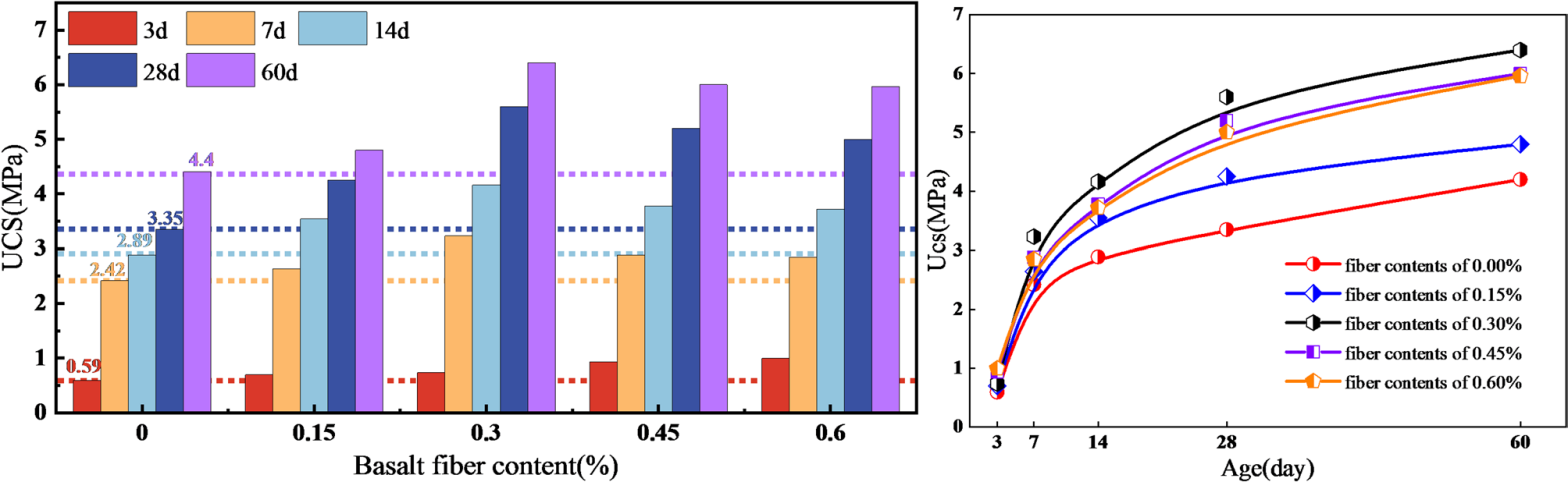
Development of uniaxial compressive strength of CGB with different basalt fiber contents.

With increasing curing age, the UCS of all specimens showed an overall upward trend, but the strength gain was strongly stage dependent, as shown in Fig. 3. The most pronounced increase occurred during the early period from 3 d to 7 d, indicating that this interval dominated the formation of the initial load-bearing skeleton. Taking the 0.30 wt% group as an example, the UCS increased from 0.74 MPa at 3 d to 3.23 MPa at 7 d, corresponding to a gain of 436.49%. After 7 d, the strength continued to increase, but the growth rate became significantly lower, suggesting that the dominant contribution of rapid early hydration gradually weakened with curing.

Under all representative curing ages, the strength response under different fiber dosages followed a typical increase-then-decrease trend, indicating the existence of a reasonable dosage window for fiber reinforcement. For both 7 d and 28 d, the 0.30 wt% group exhibited the highest UCS and the most favorable coordination with deformability. In addition, the UCS of the 0.30 wt% group at 7 d was already close to that of the fiber-free group at 28 d, further highlighting the role of basalt fiber in accelerating the formation of effective early-age load-bearing capacity.

**Fig. 4:**
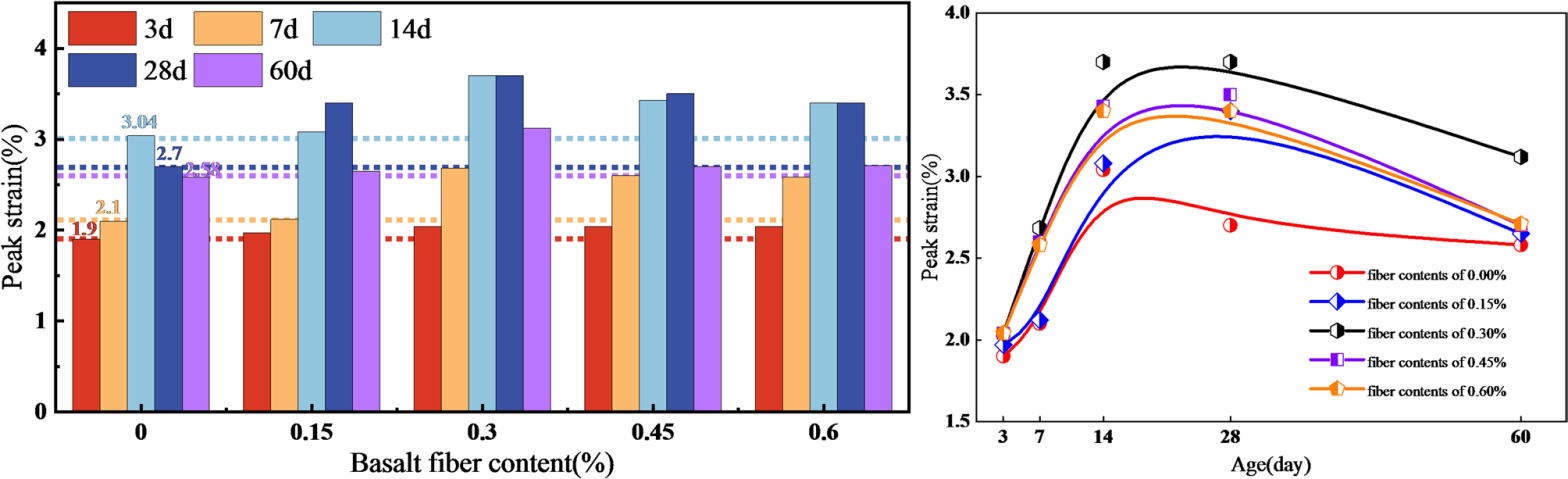
Evolution of peak strain of CGB with different basalt fiber contents under uniaxial compression.

The peak strain of both the fiber-free group and the fiber-reinforced groups increased with curing age, showing an overall trend of initial growth followed by a plateau with a slight decline. At 60 d, the peak strain of each mixture was higher than that at 7 d as shown in Fig. 4, indicating that, as the cemented structure became progressively more developed, the deformation accommodation capacity prior to failure was enhanced. Distinct differences were observed in the evolution of peak strain among mixtures with different fiber contents. The fiber-free group reached a stage maximum at 14 d (3.04%), whereas most fiber-reinforced groups with dosages of 0.15%-0.60% reached their peak at 28 d, suggesting that fiber incorporation allowed the deformation advantage to be maintained more stably into the mid-term curing stage. Notably, the 0.3 wt% group maintained a relatively high peak strain throughout the entire curing period, indicating that this dosage was beneficial not only for strength development but also for more effective improvement in pre-peak deformation compatibility and energy absorption capacity. More reliable deformation allowance can therefore be provided for early-age support and subsequent load-bearing stability of the backfill.

#### AE characteristics

**Fig. 5:**
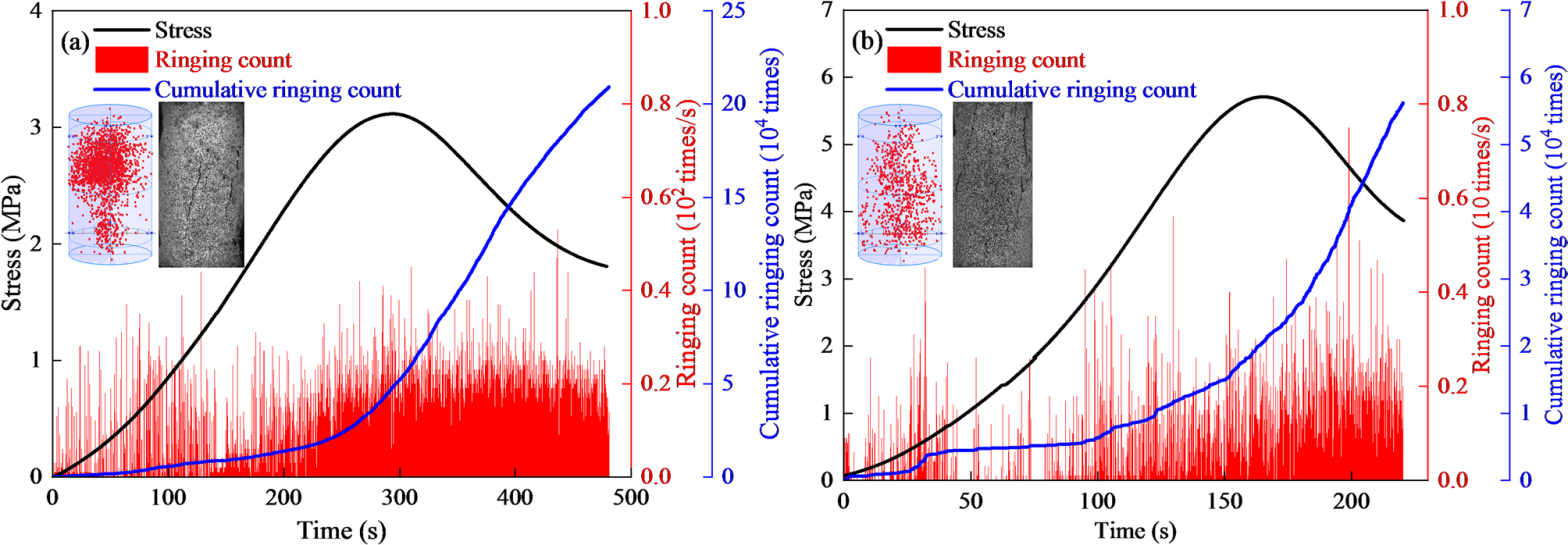
AE characteristics of backfill specimens at different early ages. (**a**) 7 d, (**b**) 28 d.

Figure 5 illustrates the evolution of stress, ring counts, and cumulative ring counts for the 7 d and 28 d specimens during uniaxial compression. The AE response can be related comparatively to the main stages of the mechanical loading process. At 7 d, ringing activity was activated earlier and remained more dispersed throughout loading, indicating that damage accumulation had already begun during the initial compaction and stable pre-peak deformation stages. This behavior is consistent with the relatively loose early-age cemented structure, in which pore closure, interfacial micro-sliding, and progressive microcrack initiation can occur continuously. The cumulative ring count reached approximately 2.17 × 10^5^.

In contrast, the 28 d specimens exhibited a more delayed and concentrated AE response. Pre-peak ring counts remained at a relatively low level for a longer period, and the cumulative curve increased slowly during the early loading stage. When the stress approached the peak region, ring counts increased sharply, indicating accelerated damage accumulation and dominant-crack formation near failure. The cumulative ring count was approximately 5.64 × 10^4^. These results suggest that curing age not only increases compressive resistance, but also shifts the damage-evolution pattern from earlier distributed activity to more concentrated AE release near peak stress. In the present study, the AE response is therefore used mainly as a comparative indicator of stage-dependent damage evolution, rather than as a rigorously normalized threshold-based criterion for crack-initiation or damage stress identification.

In a comparative sense, the AE activity can still be related to the major stages of the stress-strain response, namely initial compaction, stable pre-peak deformation, accelerated damage accumulation near peak stress, and post-peak crack coalescence. Under this framework, the 7 d specimens exhibited earlier and more dispersed AE activation, whereas the 28 d specimens showed delayed but more concentrated AE release close to the peak-stress region.

#### AE-based crack-mode evolution revealed by RA-AF analysis

AE monitoring can provide direct evidence for the initiation and propagation of microcracks in basalt-fiber-reinforced cemented gangue backfill (BFCGB) during loading. By using the combined criterion of the RA value (rise time/amplitude) and the AF value (ring counts/duration), the dominant cracking mechanism can be distinguished. Low RA together with high AF is associated mainly with tensile crack initiation, whereas high RA together with low AF more strongly indicates shear-slip-dominated failure behavior. In practice, the counts of shear and tensile cracks in rock specimens are determined using a transition line. The slope of this line is denoted as *k* and varies with material type and test conditions. Evidence indicates that suitable *k* values for rock specimens are typically in the range of 80 to 100^31^. In this study, k = 100 was adopted as a reference threshold for comparative analysis because the tested CGB exhibits a rock-like quasi-brittle response under uniaxial compression. However, this value was not independently calibrated for the present material system. Therefore, the RA-AF results are interpreted here as a semi-quantitative comparative descriptor of crack-mode evolution among different mixtures and curing ages, rather than as a fully material-specific universal classification boundary.

**Fig. 6:**
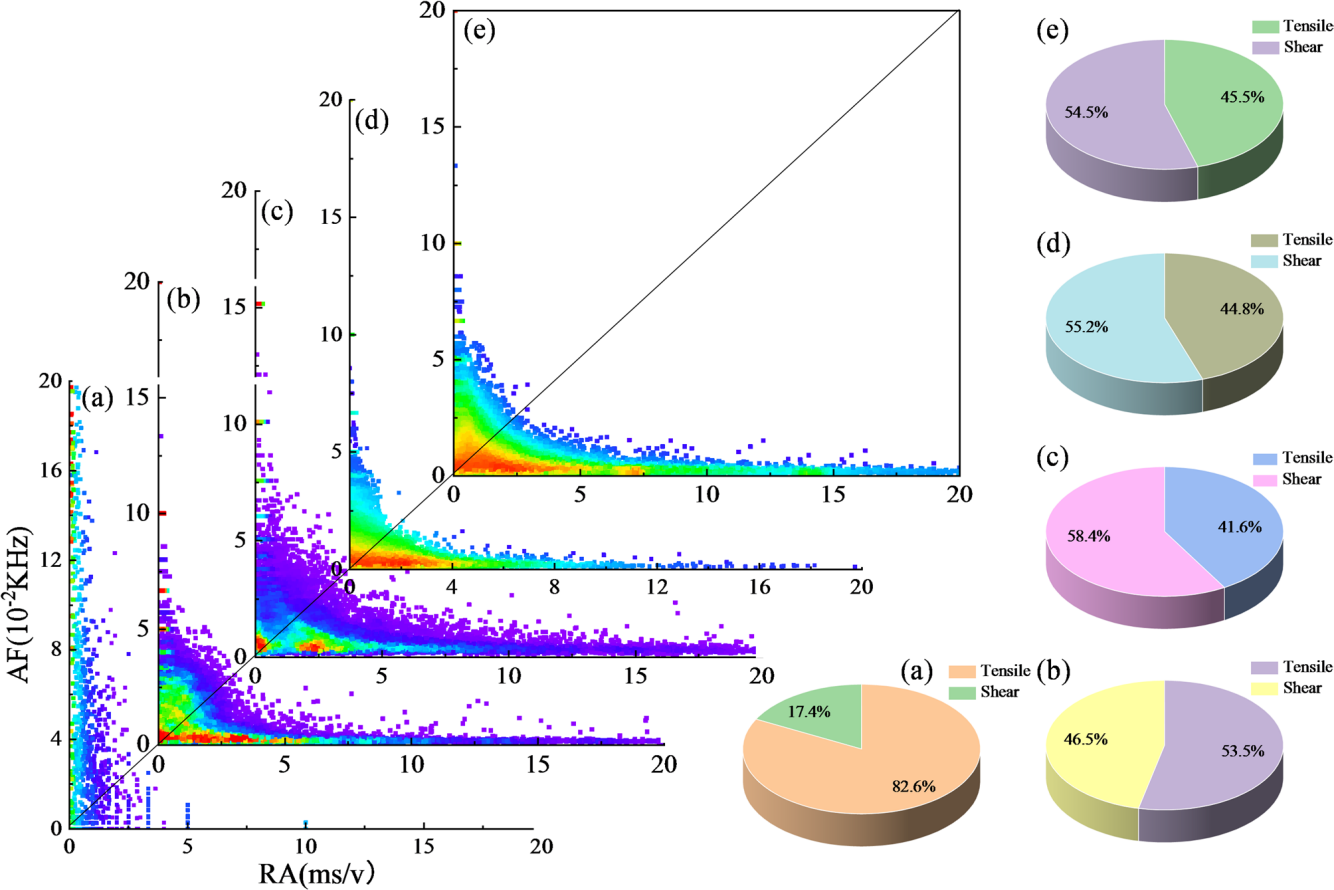
7 d RA-AF correlation distribution at each load level of basalt fiber-reinforced CGB. (**a**) 0 wt%; (**b**) 0.15 wt%; (**c**) 0.3 wt%; (**d**) 0.45 wt%; (**e**) 0.6 wt%.

At a curing age of 7 d, tensile cracking dominated the fiber-free reference group, accounting for 82.6%, which indicates a typical brittle tensile-splitting tendency when the early-age cemented structure is still insufficiently developed, as shown in Fig. 6. After basalt fibers were incorporated, a pronounced shift in cracking patterns was observed. At a fiber dosage of 0.15%, the proportion of tensile cracks decreased markedly, while the proportion of shear cracks increased by 36.1% relative to the reference group. A mixed tension-shear mechanism was therefore formed, with tensile and shear contributions of 53.5% and 46.5%, respectively. When the dosage was increased to 0.3 wt%, the shear-crack fraction further rose to 58.4%, indicating a stronger shear-dominated behavior. With further increases to 0.45% and 0.60%, the shear-crack fraction decreased slightly to 55.2% and 54.5%, but remained substantially higher than that of the fiber-free group. This trend suggests that excessive fiber addition can weaken the toughening efficiency from the optimum because dispersion becomes constrained or local interfacial defects are introduced. Nevertheless, a mixed tension-shear failure pattern was still maintained overall.

This evolution from tensile-dominated cracking to a more coordinated tension-shear damage pattern should not be interpreted to mean that a higher proportion of shear-associated cracking is always beneficial. In brittle geomaterials, localized shear failure may also represent unstable damage concentration. In the present study, the increased shear-associated AE proportion is considered favorable only when it occurs together with improved peak strain, reduced post-peak abruptness, and a more distributed crack pattern. Under these combined conditions, the increase in shear participation suggests that basalt fibers suppress the rapid coalescence of a single dominant tensile crack, promote crack deflection and branching, and enable more microcracks to dissipate energy through frictional sliding and progressive interaction. This mechanism contributes to a more damage-tolerant quasi-brittle response rather than a simple shift toward shear failure. In particular, at a dosage of 0.30 wt%, the highest shear-associated signal fraction coincided with the best strength-strain coordination, indicating that the beneficial effect was associated with distributed mixed-mode damage rather than localized shear instability. Therefore, the AE results are interpreted here in combination with the macroscopic mechanical response and crack-pattern observations, rather than being used alone as direct proof of toughening.

**Fig. 7:**
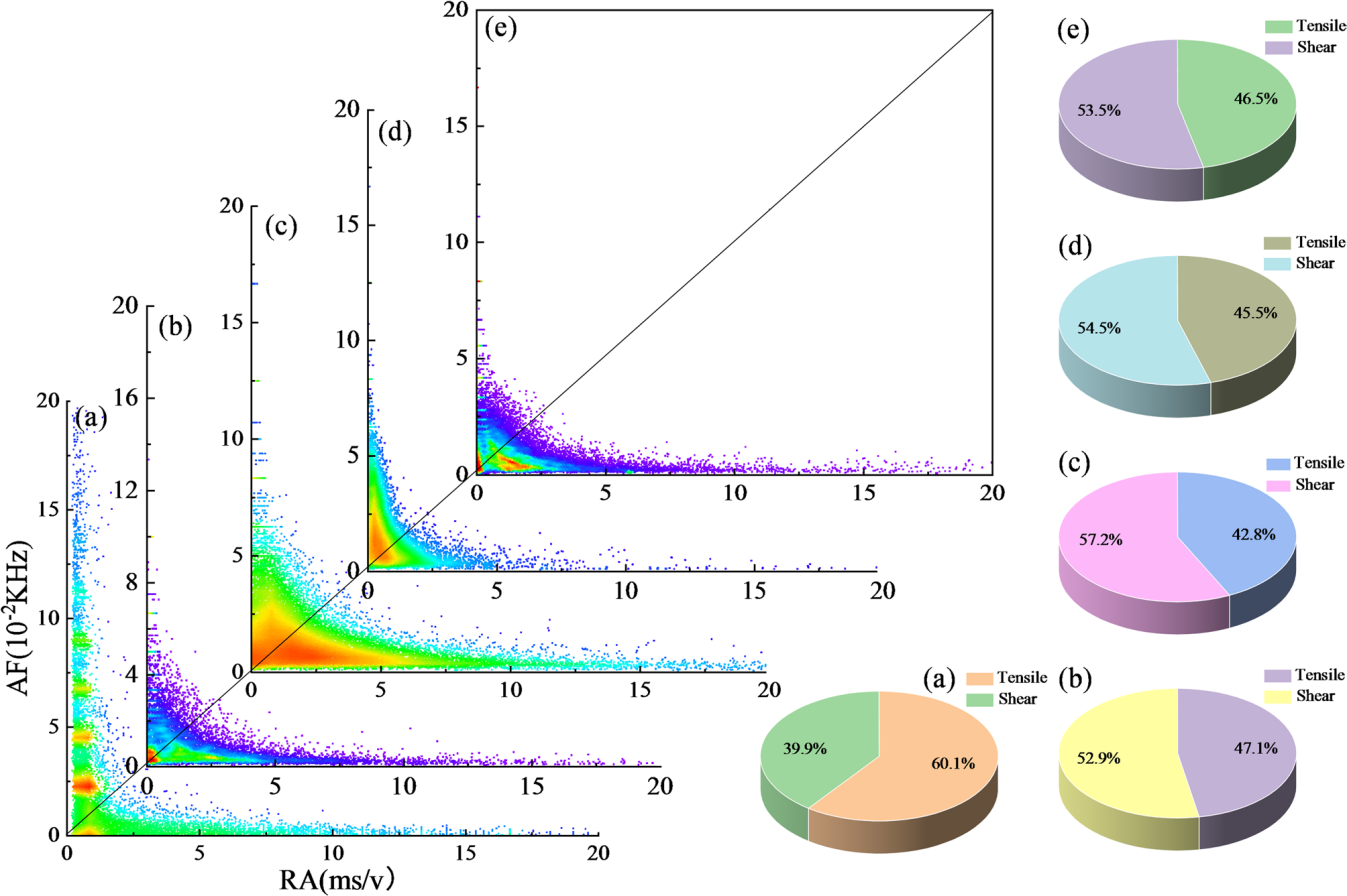
28 d RA-AF correlation distribution at each load level of basalt fiber-reinforced CGB. (**a**) 0 wt%; (**b**) 0.15 wt%; (**c**) 0.30 wt%; (**d**) 0.45 wt%; (**e**) 0.60 wt%.

When the curing age was extended to 28 d, the cracking mechanism exhibited a more pronounced matrix-controlled feature compared with that at 7 d, as shown in Fig. 7. In the fiber-free reference group, the proportion of shear cracks increased to 60.1%, indicating that, with hydration progress and structural densification, microscopic damage under uniaxial compression became more likely to concentrate along shear-sliding and frictional failure paths. The dominant cracking mechanism therefore shifted from tensile-dominated behavior at early ages to shear-dominated behavior at 28 d. After basalt fibers were added, shear cracks still dominated in all mixtures, whereas their fractions were slightly lower than that of the reference group, ranging from 52.3% to 57.2%. This trend suggests that, at the mid-term age, fiber bridging and stress redistribution could still alleviate the matrix tendency toward shear localization to some extent, and crack propagation remained more distributed. Among the fiber-reinforced mixtures, the 0.3% group exhibited the highest shear-crack fraction (57.2%), and an increase-then-decrease trend with dosage was maintained, consistent with that observed at 7 d. It should be noted that, relative to 7 d, the fiber-free group at 28 d had already entered the shear-dominated regime; therefore, the extent to which fibers regulated cracking patterns was less pronounced. The main effect was a limited modification of the degree of shear concentration rather than a fundamental reshaping of the cracking mechanism.

Taken together, the RA-AF results suggest that basalt fiber modifies the cracking tendency of CGB from predominantly tensile splitting toward a more distributed mixed-mode damage pattern. Under the unified threshold criterion adopted in this study, the 0.30 wt% group showed the most pronounced increase in shear-associated signals, which is consistent with its higher peak strain, less abrupt post-peak response, and more complex macroscopic crack morphology.

#### Macroscopic crack morphology and strain localization from surface observations and DIC

**Fig. 8:**
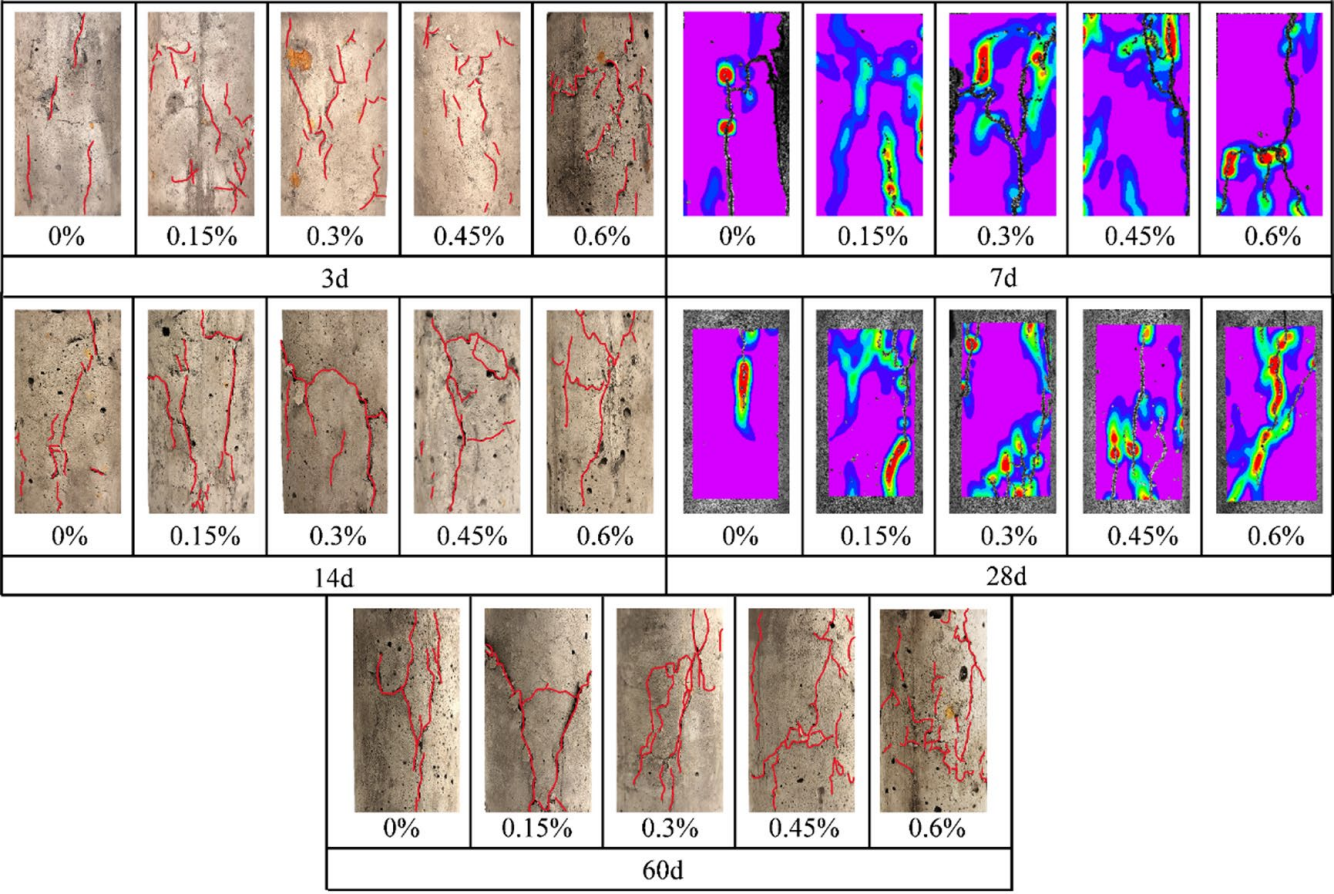
Failure patterns of basalt-fiber-reinforced backfill specimens under uniaxial compression.

When the curing age was fixed, the fiber dosage exerted a clearly staged regulation on crack morphology, as shown in Fig. 8. In the fiber-free specimens, only a few localized and non-penetrating cracks were observed near the middle part of the sample, and a typical brittle tensile-splitting feature was still exhibited. As the fiber dosage was increased to 0.15%-0.45 wt%, cracks shifted from localized concentration to a more dispersed spatial distribution. A larger number of non-penetrating mixed tension-shear microcracks were formed, whereas their individual scales remained limited. This behavior indicates that an appropriate amount of basalt fiber suppressed the rapid coalescence of a dominant crack through bridging and stress redistribution, and failure was therefore driven toward an energy-dissipating mode characterized by multiple cracks with limited penetration. When the dosage was further increased to 0.60 wt%, crack morphology changed to a penetrating oblique shear crack as the primary skeleton, accompanied by a relatively dense network of secondary cracks, and surface cracking became more pronounced. This observation suggests that a reasonable dosage window exists for fiber toughening. Excessive fiber addition can facilitate rapid crack connectivity along shear paths because of fiber agglomeration, interfacial defects, or local stress perturbations, thereby weakening the toughening effect and inducing more localized unstable failure.

With a fixed fiber dosage, crack behavior also evolved systematically with curing age. At the early stage (approximately 3–7 d), failure was dominated by dispersed, non-penetrating mixed tension-shear microcracks, which indicates that matrix strength was still developing and cracking tended to progress gradually. At the mid stage (approximately 7–28 d), as hydration products accumulated and structural continuity was enhanced, cracks evolved toward penetration. Transverse or locally penetrating shear cracks were observed together with secondary cracking, and the failure mode shifted from “dispersed microcrack domination” to “composite failure driven by a dominant crack”. At the late stage (approximately 28–60 d), the crack system became further concentrated into an obliquely penetrating shear macrocrack, indicating a more pronounced dominant-crack-controlled feature. The surface failure patterns in Fig. 8 provide visual evidence that basalt fiber modified the crack-development path from relatively concentrated tensile splitting toward a more distributed mixed-mode fracture pattern. However, the present interpretation remains mainly based on macroscopic morphological observation, and more rigorous image-based quantification, such as crack density, crack orientation statistics, or fractal descriptors, is still needed for a more objective characterization of crack geometry. Therefore, the failure-pattern analysis in this study is used as supportive visual evidence and is interpreted together with the mechanical response and AE characteristics.

At the optimal dosage of 0.30 wt%, the increase in shear-associated signals corresponded to a more dispersed oblique-crack network and reduced dominance of a single penetrating tensile crack, which is also in agreement with the higher peak strain and the less abrupt post-peak response. In this sense, the surface observations and DIC-assisted deformation features support the view that basalt fiber promotes a more distributed mixed-mode damage process rather than localized brittle splitting.

### Microstructural analysis

**Fig. 9:**
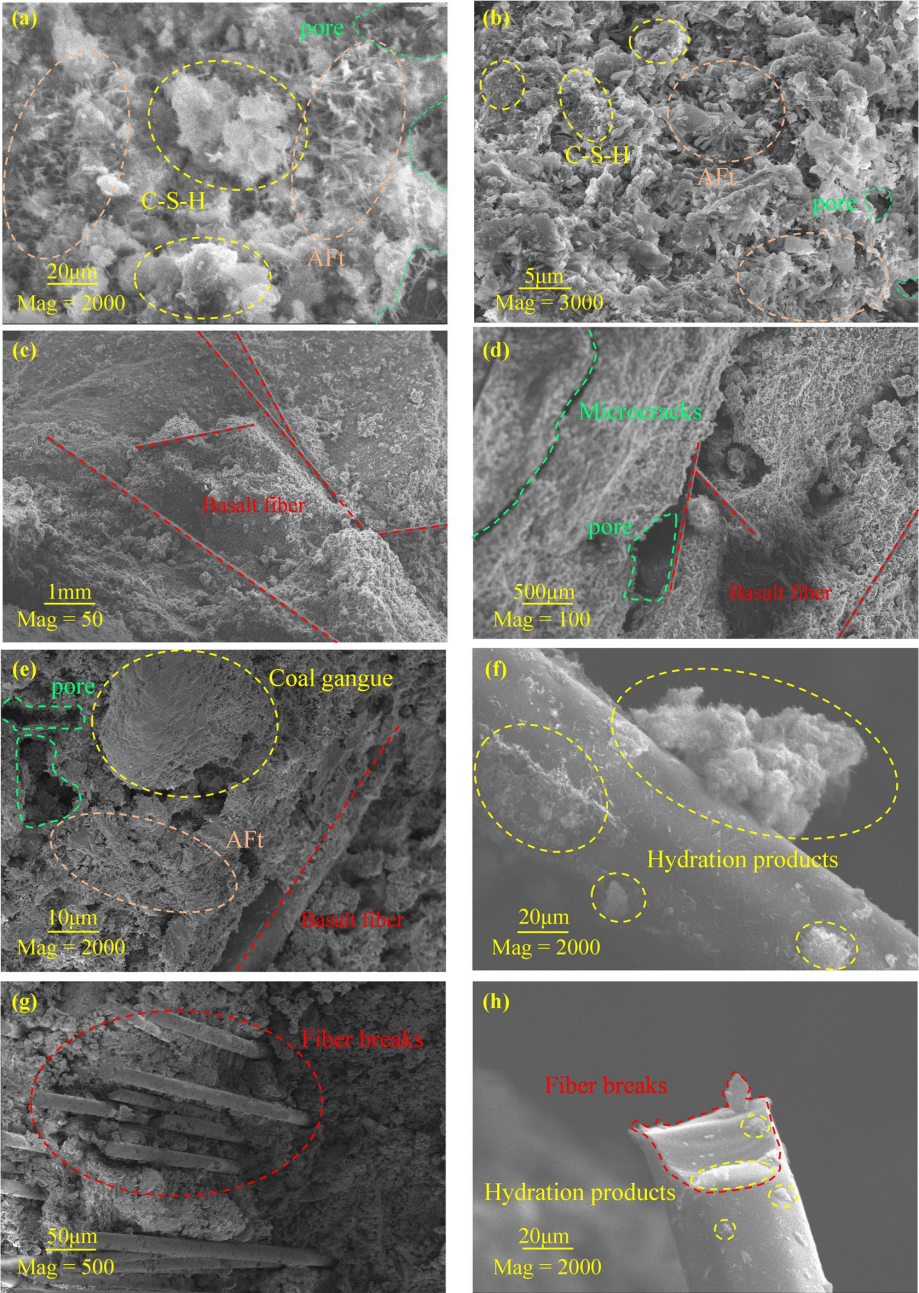
Microstructure of CGB with and without basalt fibers.

Based on the SEM observations of fracture surfaces at 7 d, Fig. 9 provides multiscale microstructural evidence for the improvement in load-bearing capacity and crack resistance of BFCGB. At the matrix scale, Fig. 9a,b shows that the reference group was characterized by flocculent C-S-H gel, needle-like ettringite (AFt), and abundant pores and micro-defects, indicating that the hydration products were present but had not yet formed a sufficiently dense and continuous load-bearing network. At the fiber-embedding scale, Fig. 9c–e shows that the 0.30 wt% specimen developed a more continuous composite skeleton in which fibers, gangue particles, and hydration products were more closely integrated. At the interfacial scale, Fig. 9f–h further reveals that a dense hydration layer dominated by C-S-H covered the fiber surface, indicating that the fiber interface acted as a favorable site for hydration-product nucleation and anchorage development. These scale-dependent observations collectively explain why the reference group remained more vulnerable to stress concentration at pores and weak interfaces, whereas the fiber-reinforced specimen exhibited improved deformation coordination and crack resistance.

In contrast, the specimen incorporating 0.3 wt% basalt fiber exhibited a more continuous and constrained microscale skeleton. Fibers were embedded more uniformly within the cemented matrix, and a denser composite structure was formed together with gangue particles and hydration products, as shown in Fig. 9c–e. More importantly, a dense layer of hydration products dominated by C-S-H was observed to cover the fiber surface, indicating that the fiber interface provided favorable sites for the nucleation and growth of hydration products. A more stable synergistic interface composed of fiber, hydration products, and matrix was therefore constructed, as shown in Fig. 9f–h. On the one hand, mechanical interlocking was enhanced by increased contact area and interfacial roughness, which reduced the likelihood of fiber debonding. On the other hand, interfacial bonding quality was improved because of material compatibility, so that load transfer from the matrix to the fibers was facilitated and stress concentration near crack tips was redistributed and dissipated more effectively. Consequently, fiber bridging and interfacial load-transfer mechanisms could participate in crack constraint and path regulation even at early ages. This observation is consistent with the superior strength and peak strain obtained at 7 d for the 0.30 wt% mixture and provides more direct material-level evidence for the concurrent enhancement of strength and toughness across macro- and micro-scales.

The reinforcing effect of basalt fiber cannot be interpreted as a simple additive outcome of physical mixing; instead, it is governed by the in-situ formation and stable development of the fiber-hydration product-matrix (F-H-M) interface. Through multiple synergistic actions, this interface improves the load-bearing behavior. When a microcrack propagates toward a fiber, strong interfacial restraint can induce crack deflection, branching, or forced detouring, thereby retarding crack coalescence. Meanwhile, more effective stress transfer and redistribution can be achieved through the interface, and local stress concentration is reduced. The denser interfacial zone can also blunt initial defects, decrease microvoid connectivity, and enhance the overall densification of the matrix. These microscale anchorage and load-transfer effects collectively explain the macroscopic performance improvement, including increased compressive strength and a shift in failure mode from single brittle tensile splitting to a more stable mixed tension-shear pattern. The dosage of 0.30 wt% is likely associated with a state in which interfacial development is more sufficient while fiber dispersion and interfacial defects remain better controlled, thereby enabling a more favorable balance between strength gain and toughness enhancement.

### Evolution analysis of the mechanical characteristics of BFCGB

(1) Model construction.

**Fig. 10:**
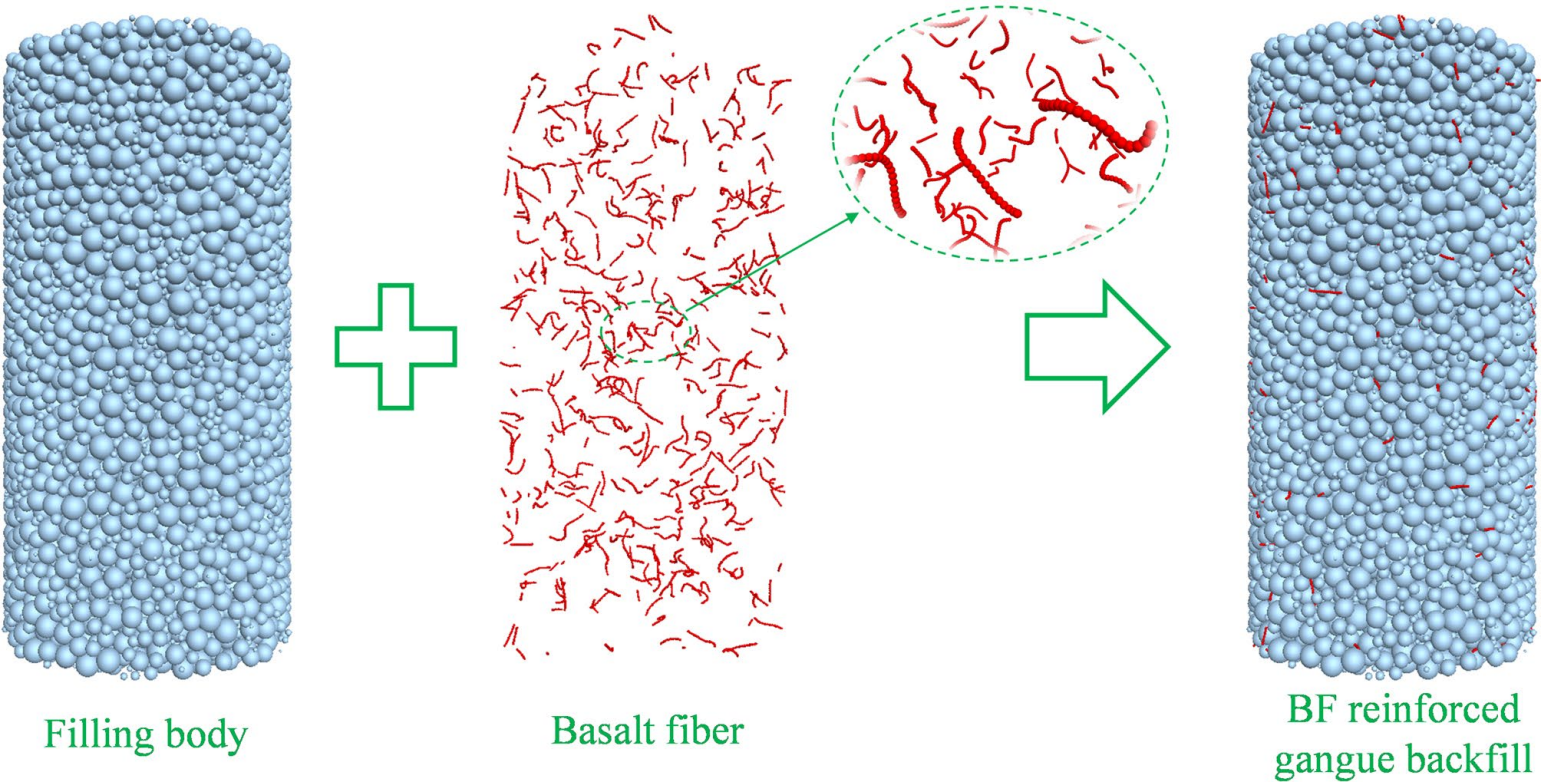
DEM workflow for representing the gangue-matrix assembly and basalt-fiber clump structure in BFCGB.

Based on the parallel-bond model, a discrete element framework was established for the present basalt-fiber-reinforced cemented gangue backfill system. The gangue-matrix assembly was represented by bonded particles with different sizes, whereas basalt fibers were constructed as clump elements in PFC. The fiber length and diameter were controlled by the number of constituent particles and the particle radius, respectively, so that the geometric characteristics of the chopped fibers used in the experiments could be approximately reflected in the numerical model. To facilitate subsequent parameter assignment and specimen generation, the clump template was first converted into ball elements and then reconverted into clump elements. The resulting workflow, shown in Fig. 10, was designed specifically to analyze how fiber dosage influences crack-network evolution and contact reorganization in BFCGB under uniaxial compression.

(2) Simulation scheme.

**Fig. 11:**
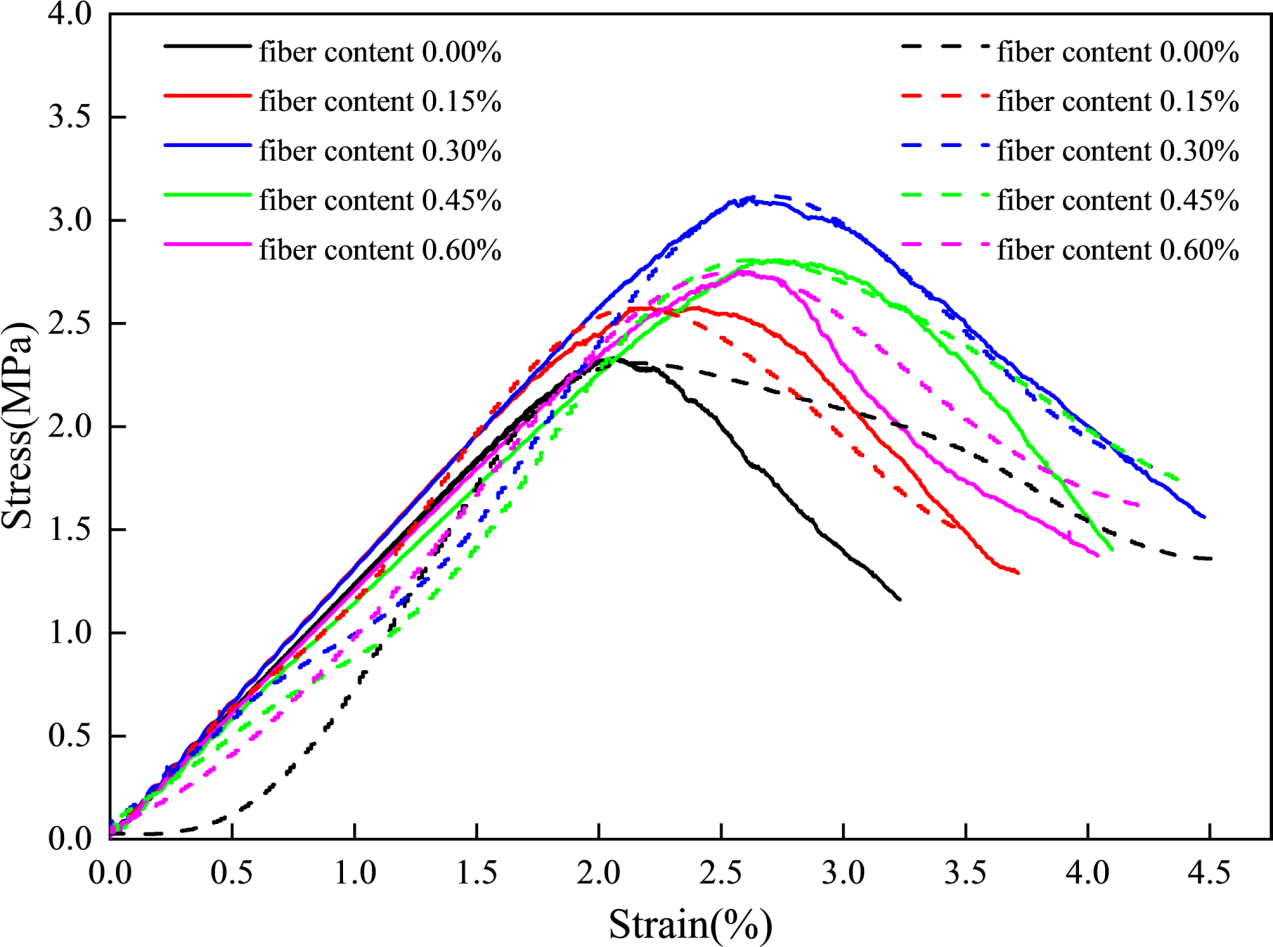
Comparison of uniaxial compression stress-strain curves obtained from laboratory tests and numerical simulations for BFCGB.

The key mechanical parameters in the bonded-particle model included the particle bulk modulus, the stiffness ratio, and the bond-related parameters controlling particle cementation, namely cohesion, tensile strength, and internal friction angle. In the present simulations, fiber dosage was represented primarily by varying the number of fiber particles in the clump template, while the remaining matrix-related contact parameters were kept constant. This simplification was adopted to isolate the first-order influence of fiber content on crack-network evolution and mechanical response, while maintaining a manageable numerical framework. Therefore, the DEM model in this study should be regarded as a reduced-order mechanistic representation rather than a full reconstruction of all coupled fiber-matrix interactions. In particular, physicochemical effects such as interfacial hydration enhancement, local bond heterogeneity, and stiffness variation induced by fiber addition were not explicitly incorporated. The numerical parameters were calibrated by back-analysis against the uniaxial compression test results. The simulation parameters are listed in Table 3, and the calibration results are shown in Fig. 11. The model reproduced the main trend of the experimental stress-strain response and was used primarily to support the interpretation of relative differences in fracture-network development among mixtures with different fiber contents. Minor discrepancies between the simulated and experimental curves are expected because the present DEM framework does not explicitly resolve full microstructural heterogeneity or all coupled fiber-matrix interfacial effects.

**Table 3:** Discrete element model parameters for cemented gangue backfill with different basalt fiber contents.

Fiber content	Fiber number	Emod (GPa)	kratio	pb_emod (GPa)	pb_kratio	pb_coh (MPa)	pb_ten (MPa)	pb_fa (°)
0.00%	0	0.24	1.5	11.2	1.5	0.85	0.62	30
0.15%	250	0.24	1.5	11.2	1.5	0.85	0.62	30
0.30%	500	0.24	1.5	11.2	1.5	0.85	0.62	30
0.45%	750	0.24	1.5	11.2	1.5	0.85	0.62	30
0.60%	1000	0.24	1.5	11.2	1.5	0.85	0.62	30

(3) Results analysis.

**Fig. 12:**
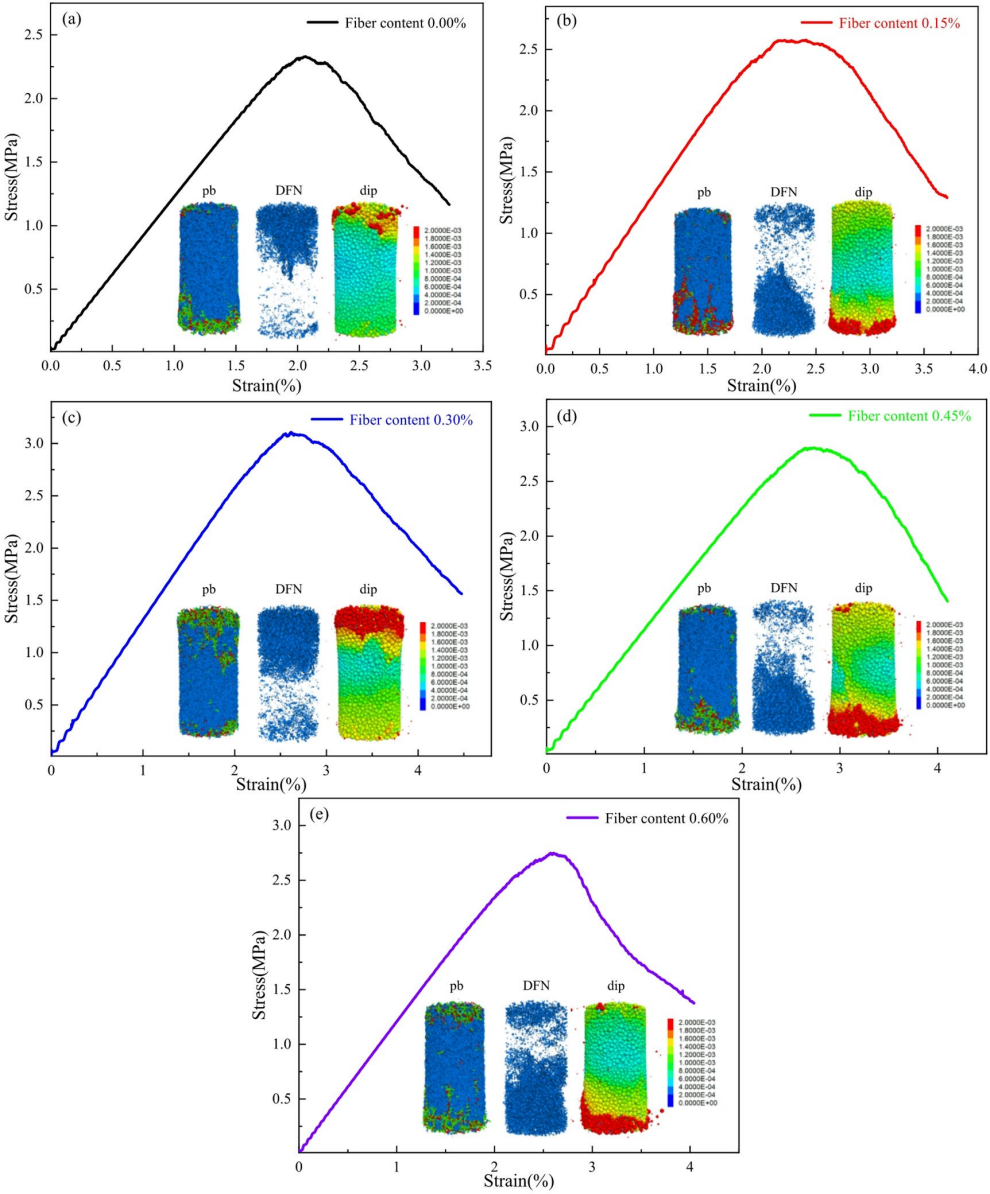
Stress-strain curves and failure characteristics of CGB with different basalt fiber contents. (**a**) 0% (fiber-free), (**b**) 0.15%, (**c**) 0.30%, (**d**) 0.45%, (**e**) 0.60%.

When the fiber dosage was increased from 0% to 0.3 wt%, the compressive strength increased from 2.33 MPa to 3.11 MPa, and the peak strain increased from 2.07% to 2.61%, indicating that an appropriate fiber content enabled an effective bridging network and synergistic load-transfer paths to be formed within the matrix. Improved deformation compatibility and energy absorption capacity before the peak were therefore obtained, as shown in Fig. 12. When the dosage was further increased to 0.6%, the compressive strength and peak strain decreased to 2.74 MPa and 2.48%, respectively, suggesting that excessive fiber addition can reduce the overall toughening efficiency because dispersion becomes constrained and local interfacial defects or stress perturbations are introduced.

This macroscopic increase-then-decrease trend is consistent with the microscale crack characteristics. The number of discrete fracture network (DFN) elements reached 21,530 at a dosage of 0.30 wt%, representing an increase of approximately 158% compared with 8,333 in the fiber-free specimen. This increase is more plausibly associated with the proliferation of distributed microcracks restrained by fibers, rather than the rapid formation of a single penetrating dominant crack. In other words, an appropriate fiber content shifted the failure process from “localized growth of a limited number of cracks” to “energy dissipation through coordinated multi-site microcracking”, which dispersed energy release in both pre-peak and post-peak stages. This provides a direct microscale explanation for the improved toughness and the evolution of failure mode toward a more stable mixed tension-shear pattern.

The higher density of distributed DFN elements at 0.30 wt% corresponds well to the increased peak strain, the less abrupt post-peak stress drop, and the more dispersed oblique crack morphology observed in the laboratory tests, indicating that both simulation and experiment support a more distributed mixed-mode damage process at the optimal fiber dosage.

**Fig. 13:**
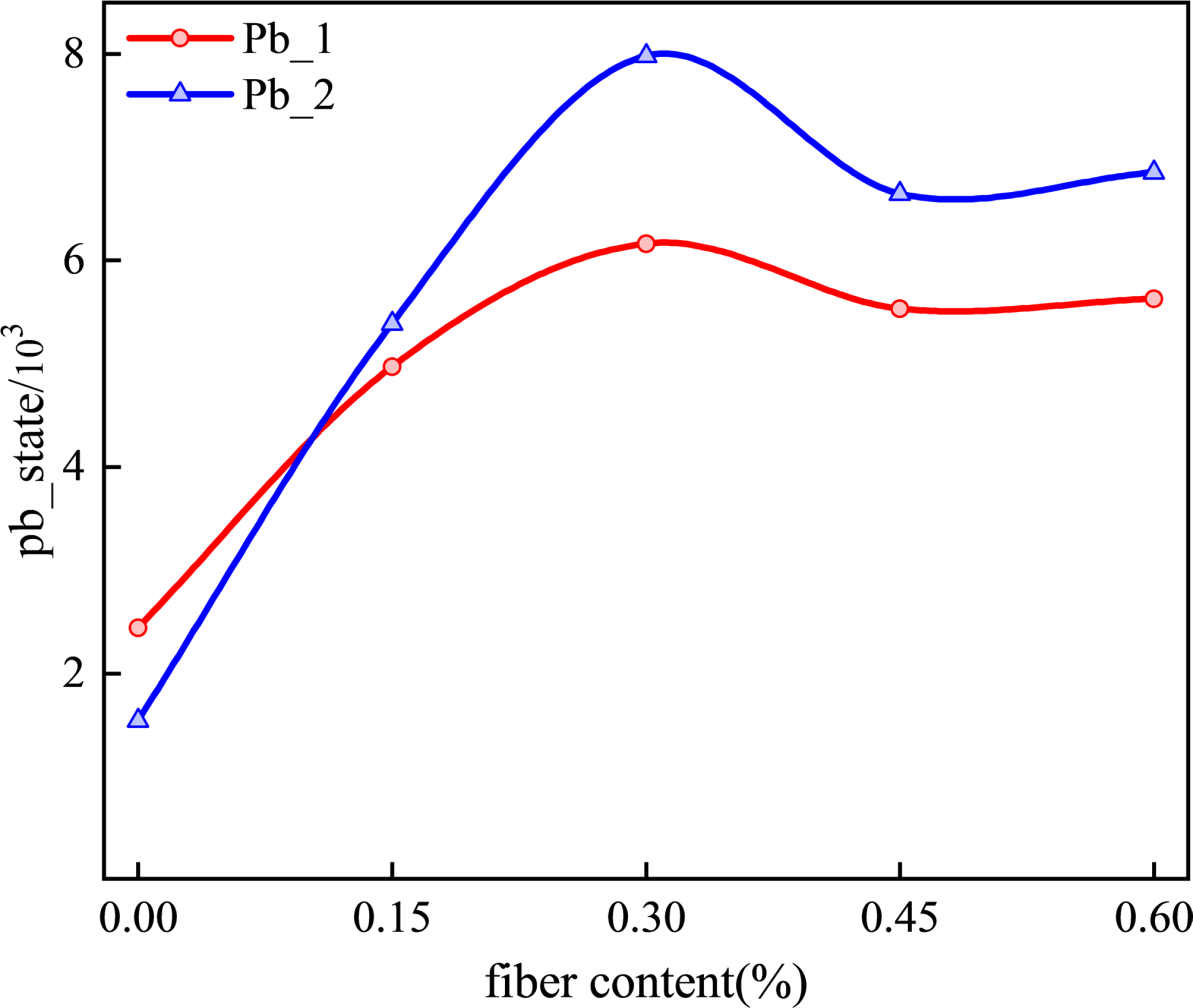
Variation in the number of different contact types with fiber dosage.

Without fiber addition, the proportion of tensile bond failures (pb_1) was markedly higher than that of shear bond failures (pb_2), indicating a microscale failure feature dominated by typical brittle tensile splitting in CGB under the tested condition, as shown in Fig. 13. After basalt fibers were incorporated, the proportion of pb_2 increased systematically, suggesting that fiber bridging and interfacial load transfer shifted the loading mode of particle contacts from tensile control toward a mechanism with more pronounced shear participation. At a dosage of 0.30 wt%, the number difference between pb_1 and pb_2 reached the maximum value of 1,820, indicating that contact-force network reconfiguration was most significant at this dosage. Load-transfer paths were therefore more likely to be redistributed in multiple directions, and energy dissipation was promoted through shear sliding and frictional mechanisms. These microscale characteristics support the concurrent improvement in macroscopic strength and deformability. When the dosage exceeded 0.3 wt%, the number of pb_2 contacts decreased. Together with the declining trend in macroscopic strength, it can be inferred that excessive fiber addition constrained dispersion and accumulated local defects, causing contact forces to re-localize in certain regions and weakening the sustained regulation of shear-dominated damage by the fibers. This contact-type evolution is also consistent with the AE results, in which the optimal fiber dosage corresponded to a higher proportion of shear-associated signals and a more distributed mixed-mode damage pattern.

## Discussion

**Fig. 14:**
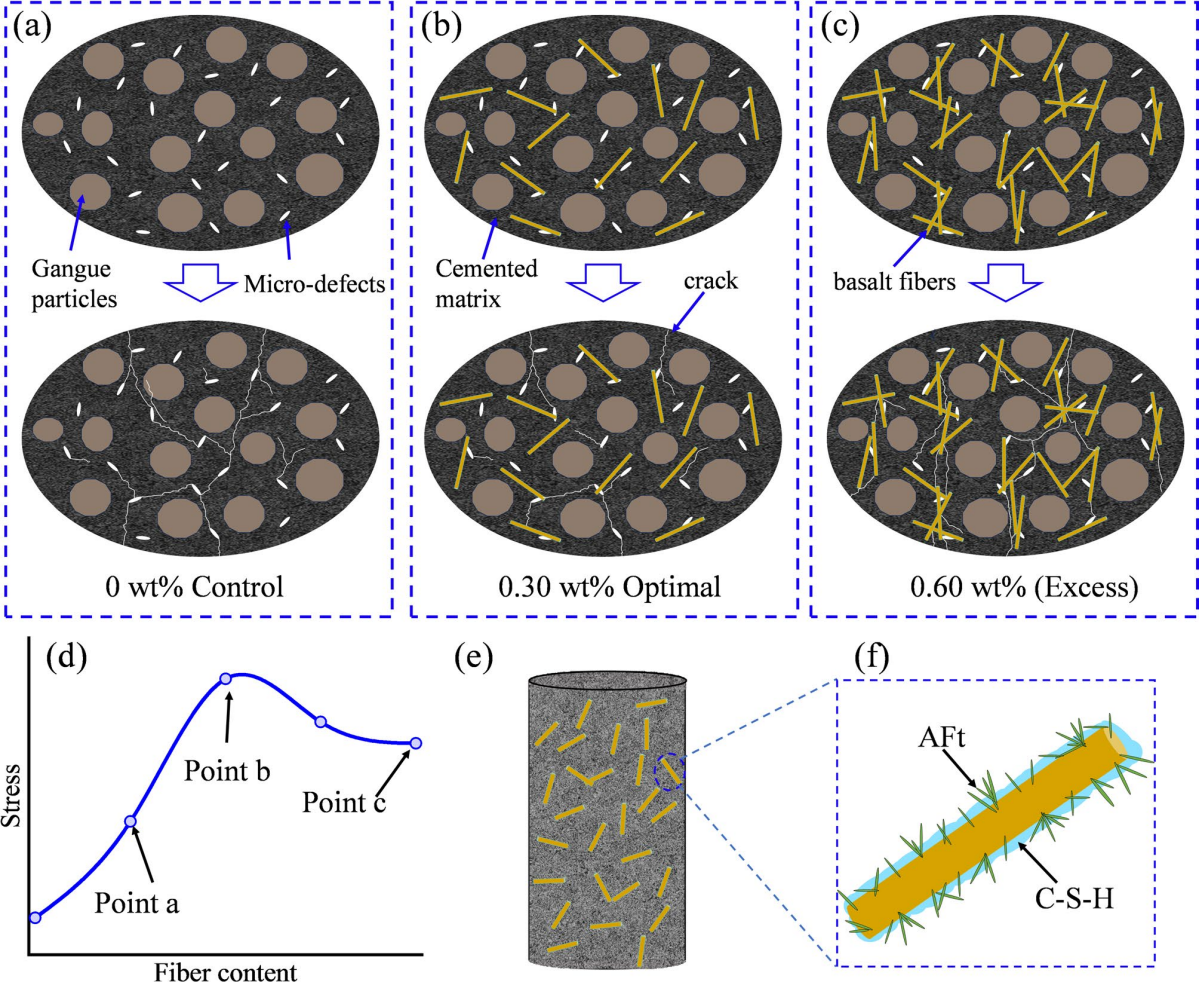
Conceptual schematic of fracture evolution in backfill with different basalt fiber contents. (**a**) 0 wt%, (**b**) 0.30 wt%, (**c**) 0.60 wt%, (**d**) Stress evolution under different fiber contents, (**e**) schematic diagram of basalt fiber filling, (**f**) F-H-M.

Mechanistically, the increase-then-decrease trend in backfill strength with increasing basalt fiber content can be interpreted as the combined outcome of fiber reinforcement and defect introduction. On this basis, Fig. 14 provides a conceptual synthesis of the proposed fracture-evolution pathway inferred from the experimental and numerical results. At low dosages, fibers can be dispersed relatively uniformly within the slurry. The fiber surface provides favorable sites for the nucleation and growth of hydration products such as C-S-H, which promotes the formation of a continuous three-phase load-bearing skeleton composed of aggregate, hydration products, and fibers, as shown in Fig. 14f. Large pores are refined and the densification of the interfacial transition zone (ITZ) is improved. Under external loading, microcrack initiation and coalescence are restrained by fiber bridging and tensile resistance, and crack paths are forced to bend and branch, as shown in Fig. 14b. As a result, both crack-initiation stress and peak stress are increased, while peak strain and energy dissipation capacity are also enhanced. Consequently, optimal strength and toughness are obtained at approximately 0.3 wt% fiber dosage.

With further increases in fiber content, slurry flowability and coating capacity are markedly reduced. Fiber entanglement and agglomeration become more severe, and some local regions cannot be fully filled by hydration products, thereby generating new defects such as fiber-cluster zones, interfacial voids, and weakly bonded bands. Under compression, these defects act as sources of stress concentration, and cracks are more likely to propagate rapidly along agglomerated regions and weak interfaces, as shown in Fig. 14c. Interfacial slip or overall debonding can therefore occur before the bridging effect is fully mobilized, which reduces the effective load-bearing cross-section and shifts failure from distributed damage to localized fracture. In this regime, the defect-introduction effect gradually exceeds the fiber-reinforcement effect, and both compressive strength and peak strain become lower than those at the optimal dosage. As a result, a mechanical response characterized by an initial increase followed by a decrease with increasing basalt fiber content is obtained, as shown in Fig. 14d.

It should also be noted that the DEM model used in this study contains a deliberate simplification. Fiber addition was represented mainly by the number of fiber elements, whereas the remaining matrix-related contact parameters were kept unchanged. As a result, the model is more suitable for revealing relative trends in crack dispersion, contact-network evolution, and failure-pattern development than for fully reproducing the coupled physicochemical interactions between fibers and the cemented gangue matrix. In particular, interfacial hydration effects, local heterogeneity, and possible fiber-induced changes in bond properties were not explicitly resolved.

From a time-dependent perspective, the uniaxial compressive strength of BFCGB increases continuously with curing age, whereas the growth rate gradually decreases. This behavior is essentially governed by the coupled effects of hydration kinetics and pore-structure evolution. At the early stage of 3–7 d, intense reactions of clinker minerals such as C_3_S occur, generating large amounts of C-S-H gel and Ca(OH)_2_. Connected pores and microcracks between the aggregate and matrix are rapidly filled, and the interfacial transition zone (ITZ) changes from loose to denser. Meanwhile, the surface of basalt fibers provides nucleation sites for hydration products, which further promotes interfacial enrichment of hydration products. As a result, the most pronounced strength gain is achieved during this period. As curing proceeds beyond 28 d, most macropores have been largely filled and free water has been substantially consumed. Pore connectivity decreases, and unhydrated particles are encapsulated by a dense C-S-H network. Ion diffusion and moisture migration paths become longer, so that hydration gradually shifts from kinetic control to diffusion control. Newly formed hydration products are then mainly involved in local void filling and crystal rearrangement, and limited benefit is provided to the overall load-bearing skeleton, leading to a flattened strength-growth curve and even a plateau. In addition, matrix densification is accompanied by drying shrinkage and internal stress accumulation, and local microcracks can develop slowly at later ages, partially offsetting the hydration-induced gain.

The fiber dosage should not be selected simply on the basis of crack-bridging capacity, because excessive addition may introduce agglomeration-related defects. Firstly, 0.30 wt% can be regarded as the near-optimal dosage, whereas 0.15 wt% provided only limited improvement and dosages of 0.45–0.60 wt% already showed signs of performance decline. Second, the interval from 3 d to 7 d should be treated as the critical early-age support window, because the major strength build-up occurred during this stage; for example, the UCS of the 0.30 wt% group increased from 0.74 MPa to 3.23 MPa during this period. Third, mixture optimization should aim not only at maximizing compressive strength, but also at improving strength-strain coordination and reducing post-peak abruptness. Under the present test conditions, the 0.30 wt% group achieved the most favorable balance, with the 28 d UCS and peak strain increasing by 67.2% and 37.0%, respectively, relative to the reference group. These values may therefore serve as a practical reference for proportion selection and early-age performance assessment in similar gangue-based backfill systems, although further verification under confined stress conditions is still necessary before they are used as generalized design criteria.

**Fig. 15:**
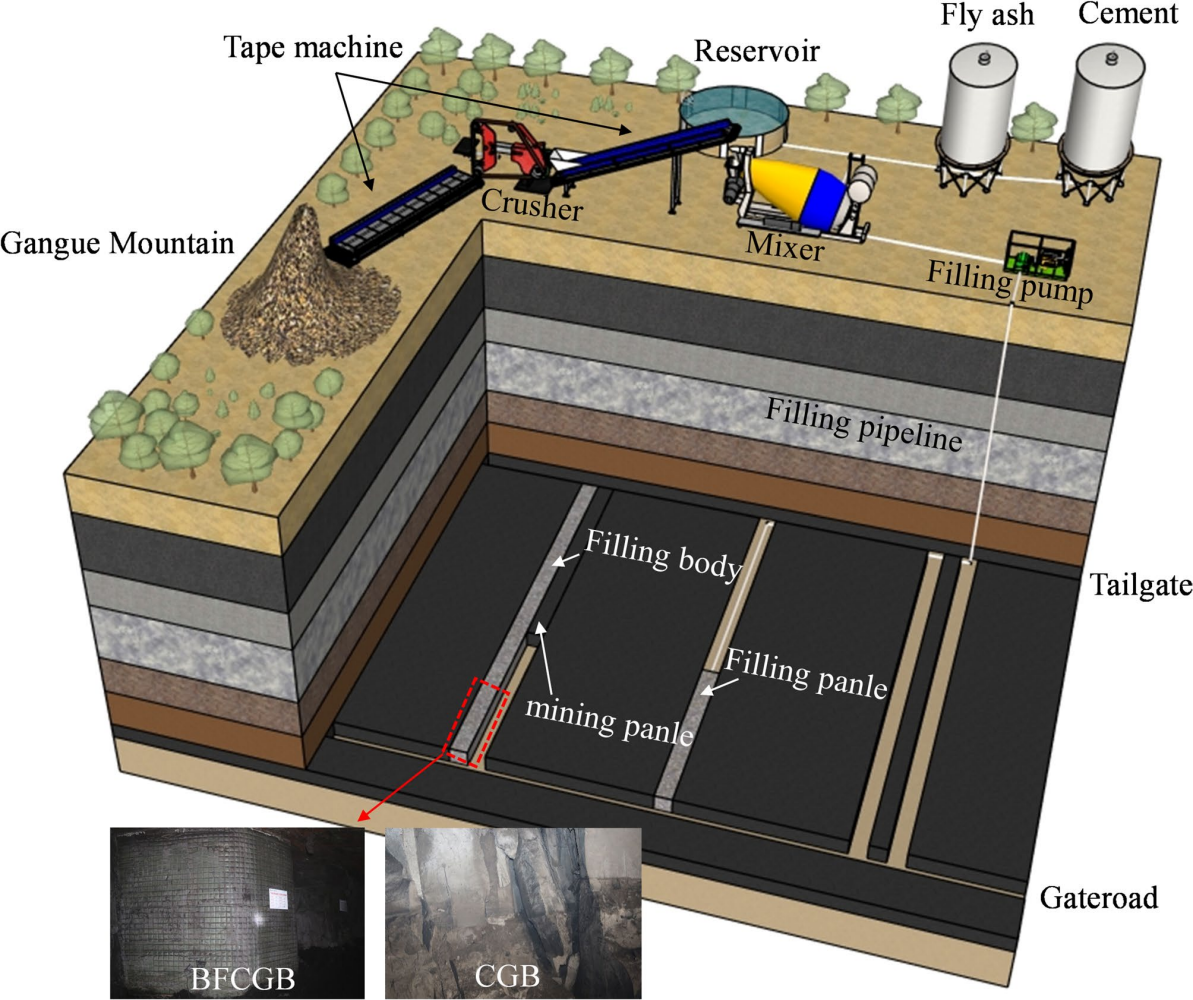
Illustrative field application of basalt-fiber-reinforced cemented gangue backfill in longwall backfilling.

On this basis, the optimized mixture proportion of BFCGB was applied in an in-situ backfilling panel to evaluate its engineering applicability under actual mining conditions. At the selected mine, continuous mining and continuous backfilling were adopted, and the goaf was supported by cemented gangue backfill prepared from gangue, fly ash, cement, and mine water. Based on the laboratory optimization results, chopped basalt fibers with a mass fraction of 0.30 wt% relative to the total solids were introduced during slurry mixing. During the field application period, the backfill body maintained good overall integrity, and no through-going tensile cracks, large-scale shear failure, or obvious instability events were observed, as shown in Fig. 15. Close contact between the backfill and the roof was also maintained throughout the observed service stage. Compared with previous conventional backfilling practice at the same mine, the roof response appeared more stable during panel advancement. These observations provide preliminary engineering-scale evidence for the feasibility and service stability of basalt-fiber-reinforced CGB under the investigated conditions. Nevertheless, because the present field validation was mainly based on engineering observation rather than systematic quantitative monitoring, further statistical comparison and long-term in-situ measurements are still required to establish rigorous engineering design guidance.

## Conclusion

Focusing on the key engineering bottlenecks of BFCGB, namely high brittleness, limited deformation coordination, and rapid post-peak instability, this study systematically clarified how basalt fibers regulate early-age load-bearing development and quasi-brittle damage evolution in gangue-based backfill from macroscopic response to microscale interfacial characteristics.

(1) Basalt fiber markedly improved the mechanical response, and an optimal dosage threshold was identified. Both strength and peak strain increased first and then decreased with increasing fiber content. The best synergy was achieved at a dosage of 0.3 wt%, at which the 28 d uniaxial compressive strength and peak strain were increased by 67.2% and 37.0%, respectively, compared with the reference group. Below this dosage, an effective bridging network was insufficiently developed; above this dosage, fiber agglomeration and pore-related defects were induced, leading to local stress concentration and a weakened reinforcing effect.

(2) The reinforcing effect was coupled with hydration progression and provided both early-age strength promotion and mid-to-late-age stability and toughness. During 3–7 d, microcrack propagation was restrained and load-transfer continuity was improved by fibers, thereby accelerating strength development. At mid-to-late ages, matrix densification was accompanied by increased brittleness; however, stable anchorage was maintained by a dense hydration-product layer at the fiber interface. Distributed micro-shear sliding and frictional energy dissipation were continuously promoted, resulting in gentler post-peak softening and enhanced deformation capacity.

(3) Failure mode was reconfigured by basalt fibers from abrupt tensile splitting toward a more distributed mixed-mode quasi-brittle damage pattern with increased shear-associated frictional dissipation. At 0.3 wt% fiber dosage, the higher proportion of shear-associated signals corresponded macroscopically to a shift from concentrated longitudinal splitting to an oblique crack network and to a less abrupt post-peak response.

(4) Interfacial bonding and microscale contact-network reorganization provided the physical basis for the synergistic enhancement of strength and toughness. SEM observations showed that an in-situ hydration-product layer dominated by C-S-H was formed on fiber surfaces, creating a composite “fiber-hydration product-matrix” interface and strengthening anchorage-based load transfer. Discrete element results indicated that an appropriate fiber content promoted crack dispersion and increased shear participation. For example, at 0.3 wt% fiber dosage, the number of DFN fractures was markedly higher than that of the reference group (21,530 vs. 8,333), which supports the macroscopic toughening and strengthening effect at the microscale.

(5) A rapid strength-gain stage was identified within the first 7 d after placement. The uniaxial compressive strength increased from 0.74 MPa to 3.23 MPa, corresponding to a rise of 436.49%. Early-age stable load bearing and resistance to instability were further enhanced by basalt fibers. Therefore, under operating conditions in which extraction is organized at approximately 7 d after backfilling, a materials-based justification for mining safety can be provided.

A basalt fiber dosage of 0.3 wt% can be regarded as the near-optimal level for performance improvement in the investigated system under the present test conditions. The risk of sudden post-peak instability was reduced through interfacial anchorage and increased shear-associated energy dissipation. However, because the present conclusions are derived mainly from uniaxial compression tests, they should be interpreted as comparative laboratory evidence rather than a complete representation of in-situ confined backfill behavior. Future work should further verify the long-term performance evolution under triaxial stress paths, dynamic loading, and coupled corrosive seepage environments.

## Data Availability

The datasets generated and/or analysed during the current study are available from the corresponding author, Dr Xiaoming Shi, upon reasonable request at shixiaoming_cumtb@163.com.
